# 1253. Optimization of empiric gram-negative infections with the combination antibiogram

**DOI:** 10.1093/ofid/ofad500.1093

**Published:** 2023-11-27

**Authors:** Anthony K Leung, Susan Harrington, Donald Dumford, Kasturi Shrestha, Bhavin Mistry, Ken wong

**Affiliations:** Cleveland Clinic Akron General, Akron, Ohio; Cleveland Clinic, Cleveland, Ohio; Cleveland Clinic Akron General, Akron, Ohio; Cleveland Clinic Akron General, Akron, Ohio; Cleveland Clinic Akron General, Akron, Ohio; Cleveland Clinic Akron General, Akron, Ohio

## Abstract

**Background:**

Anti-pseudomonal beta-lactam drugs such as aztreonam (AZR), cefepime (CEF), piperacillin-tazobactam (P/T), meropenem (MER) are drugs used for empiric gram-negative (GN) infections. They are used with other drugs as empiric combination therapy (CT) for patients with sepsis. The antibiogram is used to provide data for empiric therapy and gauge susceptibility pattern shifts over time. Combining different classes of drugs might enhance the susceptibility to various pathogens. This project assessed which empiric combination of drugs will yield optimal coverage for various GN pathogens from different sources.

**Methods:**

In 2022 antimicrobial susceptibility data from 8288 isolates from blood, respiratory, wound and urine sources were collected and analyzed. Percent susceptibility was calculated for 11 gram-negative species with >30 isolates. Susceptibility testing was done by BD Phoenix™ automated identification and susceptibility testing system. A threshold of >90% susceptibility was used to indicate optimal coverage for the antimicrobial agents. CT is calculated as the % susceptible to either of two antibiotics.

**Results:**

In respiratory sources AZR, CEF and P/T were the lowest at 73%, 83% and 77% susceptible. MER had the best overall coverage for all specimen types. Adding either an aminoglycoside (AG) or fluoroquinolone (FQ) increased susceptibility in all sources. AGs had a slightly better coverage than FQs. For blood isolates > 90% susceptibility was attained for single antibiotics without combination. CT was often required to achieve optimal coverage for respiratory sources, specifically for *Pseudomonas*, *Enterobacter*, *E coli*, *Klebsiella* and *Proteus* species.

**Figure 1.**
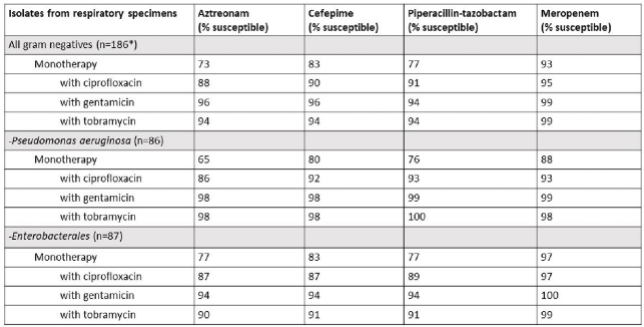
Combination antibiogram for beta-lactam agents for gram-negative respiratory isolates as monotherapy or in combination with ciprofloxacin, gentamicin, or tobramycin. *All gram-negative isolates included Serratia and Providencia species that were not included in the subgroups.

**Conclusion:**

Using > 90% susceptibility as cut-off, monotherapy AZR and P/T are suboptimal for empiric coverage for certain GN respiratory infections. Blood specimens may not require CT. Adding either AGs or FQs enhanced susceptibility coverage to all tested pathogens but not uniformly. AGs enhancement was slightly better than FQs. Respiratory infections with *Pseudomonas*, *Enterobacter cloacae*, *E coli*, *Proteus* and *Klebsiella* species all showed enhanced coverage with CT especially for P/T and AZR. Every hospital should create a combination antibiogram to gain insight into whether empiric coverage with CT is needed.

**Disclosures:**

**All Authors**: No reported disclosures

